# Supplementary material for: Effects of environmental hypoxia and hypercarbia on ventilation and gas exchange in Testudines
Source: PeerJ. 2018 Jul 11;6:e5137. doi: 10.7717/peerj.5137 (PMC6045925; doi:10.7717/peerj.5137)
Supplement: Table S1 [file peerj-06-5137-s001.docx]

**Supplementary Table 1**: Research papers presenting data on ventilatory parameters of Cryptodira and Pleurodira exposed to hypoxia or hypercarbia. Studies exposing the animals to anoxia have not been included here. Each species family is given in parenthesis.

| **Species** | **Reference(s)** | |
| --- | --- | --- |
|  | Hypoxia | Hypercarbia |
| Cryptodira |  |  |
| *Chelonia mydas*  (Cheloniidae) | Jackson, Kraus & Prange, 1979; Jackson, 1985 | Jackson, Kraus & Prange, 1979; Jackson, 1985 |
| *Chelydra serpentina*  (Chelydridae) | Boyer, 1963, 1966; Frische, Fago & Altimiras, 2000; West, Smits & Burggren, 1989 | West, Smits & Burggren, 1989 |
| *Chrysemys picta*  (Emydidae) | Funk & Milsom, 1987; Glass, Boutilier & Heisler, 1983; Milsom & Chan, 1986 | Jackson, Singer & Downey, 1991; Milsom & Jones, 1979, 1980; Milsom & Chan, 1986; Silver & Jackson, 1985 |
| *Dermochelys coriacea*  (Dermochelyidae) | Price et al., 2007 | Price et al., 2007 |
| *Gopherus polyphemus*  (Testudinidae) | Ultsch & Anderson, 1988 | Ultsch & Anderson, 1988 |
| *Lepidochelys olivacea*  (Cheloniidae) | Price et al., 2007 | Price et al., 2007 |
| *Terrapene carolina*  (Emydidae) | Altland & Parker, 1955; Ultsch & Anderson, 1988 | Ultsch & Anderson, 1988 |
| *Testudo horsfieldii*  (Testudinidae) | Benchetrit, Armand & Dejours, 1977 | Benchetrit & Dejours, 1980 |
| *Testudo pardalis*  (Testudinidae) | Burggren, Glass & Johansen, 1977; Glass, Burggren & Johansen, 1978 | Burggren, Glass & Johansen, 1977; Glass, Burggren & Johansen, 1978 |
| *Trachemys scripta*  (Emydidae) | Frankel et al., 1969; Herman & Smatresk, 1999; Hicks & Wang, 1999; Hitzig & Nattie, 1982; Jackson & Schmidt-Nielsen, 1966; Jackson, 1973; Vitalis & Milsom, 1986; Johnson, Krisp & Bartman, 2015; Lee & Milsom, 2016; Reyes & Milsom, 2009 | Hitzig & Nattie, 1982; Herman & Smatresk, 1999; Jackson, Palmer & Mead, 1974; Vitalis & Milsom, 1986; Johnson & Creighton, 2005; Reyes & Milsom, 2009 |
| Pleurodira |  |  |
| *Pelomedusa subrufa*  (Pelomedusidae) | Burggren, Glass & Johansen, 1977; Glass, Burggren & Johansen, 1978 | Burggren, Glass & Johansen, 1977; Glass, Burggren & Johansen, 1978 |
| *Phrynops geoffroanus*  (Chelidae) | Cordeiro, Abe & Klein, 2016 | Cordeiro, Abe & Klein, 2016 |
| *Podocnemis unifilis*  (Podocnemididae) | Cordeiro, Abe & Klein, 2016 | Cordeiro, Abe & Klein, 2016 |

**References**

Altland PD, Parker M. 1955. Effects of hypoxia upon the Box Turtle. Am. J. Physiol. 180, 421-427.

Benchetrit G, Armand J, Dejours P. 1977. Ventilatory chemoreflex drive in the tortoise, *Testudo horsfieldi*. Resp. Physiol. 31, 183-191.

Benchetrit G, Dejours P. 1980. Ventilatory CO_2_ drive in the tortoise *Testudo horsfieldi*. J. Exp. Biol. 87, 229-236.

Boyer DR 1963. Hypoxia: Effects on heart rate and respiration in the snapping turtle. Science 140, 813-814.

Boyer DR. 1966. Comparative effects of hypoxia on respiratory and cardiac function in reptiles. Physiol. Zool. 39, 307-316.

Burggren WW. 1975. A quantitative analysis of ventilation tachycardia and its control in two chelonians, *Pseudemys scripta* and *Testudo graeca*. J. Exp. Biol. 63, 367-380.

Burggren WW, Glass ML, Johansen K. 1977. Pulmonary ventilation: perfusion relationships in terrestrial and aquatic chelonian reptiles. Can. J. Zool. 55, 2024-2034.

Cordeiro TEF, Abe AS, Klein W. 2016. Ventilation and gas exchange in two turtles: *Podocnemis unifilis* and *Phrynops geoffroanus* (Testudines: Pleurodira). Respir. Physiol. Neurobiol. 224, 125-131.

Frankel HM, Spitzer A, Blaine J, Schoener EP. 1969. Respiratory response of turtles (*Pseudemys scripta*) to changes in arterial blood gas composition. Comp. Biochem. Physiol. 31, 535-546.

Frische S, Fago A, Altimiras J. 2000. Respiratory responses to short term hypoxia in the snapping turtle, *Chelydra serpentina*. Comp. Biochem. Physiol. Part A. 126, 223-231.

Funk GD, Milsom WK. 1987. Changes in ventilation and breathing pattern produced by changing body temperature and inspired CO_2_ concentration in turtles. Respir. Physiol. 67, 37-51.

Glass ML, Burggren WW, Johansen K. 1978. Ventilation in an aquatic and a terrestrial chelonian reptile. J. Exp. Biol. 72, 165-179.

Glass ML, Boutilier RG, Heisler N. 1983. Ventilatory control of arterial PO_2_ in the turtle *Chrysemys picta belli*: Effects of temperature and hypoxia. J. Comp. Physiol. 151, 145-153.

Herman JK, Smatresk NJ. 1999. Cardiorespiratory response to progressive hypoxia and hypercapnia in the turtle *Trachemys scripta*. J. Exp. Biol. 202, 3205–3213.

Hicks JW, Wang T. 1999. Hypoxic hypometabolism in the anesthetizes turtle, *Trachemys scripta*. American Physiological Society. 277, R18-R23.

Hitzig BM, Nattie EE. 1982. Acid-base stress and central chemical control of ventilation in turtles. J. Appl. Physiol. 53, 1365-1370.

Jackson DC. 1973. Ventilatory response to hypoxia in turtles at various temperatures. Respir. Physiol. 18, 178-187.

Jackson DC. 1985. Respiration and respiratory control in the green turtle, *Chelonia mydas*. Copeia, 1985, 664-671.

Jackson DC, Schmidt-Nielsen K. 1966. Heat production during diving in the fresh water turtle, *Pseudemys scripta*. J. Cellular Physiol. 67, 225-231.

Jackson DC, Singer JH, Downey PT. 1991. Oxidative cost of breathing in the turtle *Chrysemys picta bellii*. Amer. J. Physiol. Reg. Int. Comp. Physiol 30, R1325-R1328

Jackson DC, Palmer SE, Meadow WL. 1974. The effects of temperature and carbon dioxide breathing on ventilation and acid-base status of turtles. Respir. Physiol. 20, 131-146.

Jackson DC, Kraus DR, Prange HD. 1979. Ventilatory response to inspired CO_2_ in the sea turtle: effects of body size and temperature. Resp. Physiol. 38, 71-81.

Johnson SM, Creighton RJ, 2005. Spinal cord injury-induced changes in breathing are not due to supraspinal plasticity in turtles (*Pseudemys scripta*). Am. J. Physiol. Regul. Integr. Comp. Physiol. 289, R1550–R1561.

Johnson SM, Krisp AR, Bartman ME. 2015. Hypoxia switches episodic breathing to singlet breathing in red-eared slider turtles (*Trachemys scripta*) via a tropisetron-sensitive mechanism. Resp. Physiol. Neurobiol. 207, 48–57.

Lee SY, Milsom WK. 2016. The metabolic cost of breathing in red-eared sliders: An attempt to resolve an old controversy. Respir. Physiol. Neurobiol. 224, 114–124.

Milsom WK, Jones DR. 1979. Pulmonary receptor chemosensivity and the ventilatory response to inhaled CO_2_ in the turtle. Respir. Physiol. 37, 101-107.

Milsom WK, Jones DR. 1980. The role of vagal afferent information and hypercapnia in control of the breathing pattern in Chelonia. J. Exp. Biol. 87, 53-63.

Milsom WK, Chan P. 1986. The relantioship between lung volume, respiratory drive and breathing pattern in the turtle, *Chrysemys picta*. J. Exp. Biol. 120, 233-247.

Price ER, Paladino FV, Strohl KP, Pilar Santidrián T, Klann K, Spotila JR. 2007. Respiration in neonate sea turtles. Comp. Biochem. Physiol. A 146, 422–428.

Reyes C, Milsom WK. 2009. Daily and seasonal rhythms in the respiratory sensitivity of red-eared sliders (*Trachemys scripta elegans*). J. Exp. Bio. 212, 3339-3348.

Silver RB, Jackson DC. 1985. Ventilatory and acid-base response to long-term hypercapnia in the freshwater turtle, *Chrysemys picta bellii*. J. Exp. Biol. 144, 661-672.

Ultsch GR, Anderson JF. 1988. Gas exchange during hypoxia and hypercarbia of terrestrial turtles: A comparison of a fossorial species (*Gopherus polyphemus*) with a sympatric nonfossorial species *(Terrapene carolina*). Physiol. Zool. 61, 142-152.

Vitalis TZ, Milsom WK. 1986b. Mechanical analysis of spontaneous breathing in the semi-aquatic turtle, *Pseudemys scripta*. J. Exp. Biol. 125, 157-171.

West NH, Smits AW, Burggren WW. 1989. Factors terminating nonventilatory periods in the turtle, *Chelydra serpentina*. Respir. Physiol. 77, 337-350.
